# Parallel Genome-Wide Fixation of Ancestral Alleles in Partially Outcrossing Experimental Populations of *Caenorhabditis elegans*

**DOI:** 10.1534/g3.114.012914

**Published:** 2014-07-01

**Authors:** Christopher H. Chandler

**Affiliations:** Department of Biological Sciences, SUNY Oswego, Oswego, New York 13126

**Keywords:** experimental evolution, selective sweep, compensatory adaptation, sex determination, *tra-2*

## Abstract

Experimental evolution studies, coupled with new advances in DNA sequencing technology, have become a powerful tool for exploring how populations respond to selection at the genomic level. Recent experiments in microbes typically have found evidence for multiple novel mutations, which are usually fixed. In contrast, in animal model systems, evolutionary responses seem to involve more modest changes in the frequencies of pre-existing alleles, probably because these populations outcross and are usually initialized with greater levels of standing variation. In this experiment, I used whole-genome resequencing to estimate allele frequencies and look for novel substitutions in experimentally evolved populations of *Caenorhabditis elegans*. These populations were founded with a fixed pair of deleterious mutations introgressed into multiple wild genetic backgrounds and allowed to evolve for 50 generations with a mixed mating system. There is evidence for some recombination between ancestral haplotypes, but selective sweeps seem to have resulted in the fixation of large chromosomal segments throughout most of the genome. In addition, a few new mutations were detected. Simulations suggest that strong selection and low outcrossing rates are likely explanations for the observed outcomes, consistent with earlier work showing large fitness increases in these populations over 50 generations. These results also show clear parallels to population genetic patterns in *C. elegans* in nature: recent selective sweeps, high linkage disequilibrium, and low effective recombination rates. Thus, the genomic consequences of selection depend heavily on the biology of the organism in question, including its mating system and levels of genetic variation.

Experimental evolution has long been a powerful tool for biologists (*e.g.*, Malmberg 1977; Rose 1984; Dodd 1989; Lenski *et al.* 1991; Rice 1992). Only recently, however, has the widespread adoption of high-throughput DNA sequencing technology made “evolve-and-resequence” a feasible experimental strategy (*e.g.*, Burke *et al.* 2010; Turner *et al.* 2011; Remolina *et al.* 2012). In this approach, populations of a model organism are reared in the laboratory for a number of generations, usually using an experimentally manipulated selective agent. After experimental evolution, whole-genome resequencing is used to identify genetic loci responding to selection. This experimental design has the potential to shed light on a number of important evolutionary questions, including the repeatability of the genetic basis of evolutionary change and how selection affects variation at linked sites (Kawecki *et al.* 2012; Gray and Cutter 2014).

To date, evolve-and-resequence studies have largely fallen along two ends of a continuum. On one end, populations are initiated with a single isogenic progenitor, relying entirely on *de novo* mutations to drive adaptation, and typically lacking genetic recombination. This design is typical of microbial experiments (*e.g.*, Araya *et al.* 2010; Charusanti *et al.* 2010). Such studies, in general, have found a handful of novel substitutions in adapted populations, with mutations in similar genes or pathways often occurring convergently in independent experimental replicates (Araya *et al.* 2010; Charusanti *et al.* 2010; Comas *et al.* 2011; Dettman *et al.* 2012).

On the other end of this continuum are studies initialized with a considerable degree of standing genetic variation and with obligately outcrossing populations, typically using animal systems (to date, mostly *Drosophila melanogaster*). These organisms typically also have larger genomes, potentially offering more mutational targets for adaptation, although the population mutation rate (N_e_μ) often is smaller in absolute magnitude because of the population sizes used. Although the repeatability of genetic changes is difficult to assess in these studies because they often identify candidate loci by looking for shared patterns across replicates, there usually are multiple loci showing a common signal (Burke *et al.* 2010; Turner *et al.* 2011; Zhou *et al.* 2011; Orozco-Terwengel *et al.* 2012; Remolina *et al.* 2012; Turner and Miller 2012; Tobler *et al.* 2014), suggesting that some degree of repeatability is common. However, in contrast to microbial studies, animal studies typically find polygenic responses involving ancestral alleles rather than novel mutations and fewer fixed sites. One notable exception among these animal studies involved the nematode worm *Caenorhabditis elegans*, in which a handful of novel substitutions became fixed, likely due to selective sweeps (Estes and Lynch 2003; Denver *et al.* 2010). However, this study adopted a setup similar to microbial experiments, involving isogenic, self-fertilizing populations. Thus, the differences in outcomes between microbial and animal studies are likely explained by both their experimental designs and the biology of the study organisms.

Although the two endpoints of this continuum may provide reasonable laboratory models for many organisms, there are of course others that fall somewhere in between. *C. elegans*, for instance, is androdioecious, with primarily self-fertilizing hermaphrodites and outcrossing males. In natural populations, male frequencies and outcrossing rates are low (Barrière and Félix 2005, 2007). Genetic diversity in this species is also low, and there are large blocks of strong linkage disequilibrium, even extending across separate chromosomes (Rockman and Kruglyak 2009; Andersen *et al.* 2012). Patterns of genetic variation in natural populations provide evidence for recent and rapid selective sweeps (Andersen *et al.* 2012). In addition, effective population outcrossing rates estimated from population genetic data are orders of magnitude lower than those estimated directly from the occurrence of males and heterozygotes in nature, suggesting outbreeding depression or selection against recombinant genotypes, probably due to epistasis (Barrière and Félix 2007; Dolgin *et al.* 2007; Rockman and Kruglyak 2009). (However, some laboratory studies suggest that outcrossing may facilitate adaptation to novel or changing environments, at least in some situations; Morran *et al.* 2009; 2011; Teotonio *et al.* 2012). Based on these observations, selective sweeps extending across large chromosomal segments also might be expected to occur in androdioecious experimental populations of *C. elegans*, even in the presence of standing variation. If these sweeps involve ancestral variants rather than novel mutations, then repeatable outcomes are also predicted.

In this work, I follow up on an earlier experimental evolution study in *C. elegans* to test these predictions. These populations were fixed for a pair of mutations rendering sex determination temperature-sensitive but introgressed into multiple wild genetic backgrounds, so they were founded with standing genetic variation (Chandler *et al.* 2012). They also exhibited a mixed mating system, with some degree of facultative outcrossing (at least initially), allowing for some recombination between divergent haplotypes. Ten populations were evolved at each of two intermediate temperatures for 50 generations. In these environments, the temperature-sensitive mutant genotype exhibits dramatically deleterious effects: fecundity is reduced by up to 75%, with as many as half the worms in the founder populations being infertile or sterile (Chandler *et al.* 2009; 2012). Ancestral worms at these temperatures also exhibit obvious intersexuality in their tail morphology, which is sexually dimorphic in this species. After 50 generations of experimental evolution, however, fertility rates and tail morphology have recovered in some populations to near wild-type levels, suggesting that selection has favored compensatory alleles suppressing these deleterious mutant phenotypes. However, transcript levels of five key sex-determining genes cannot explain this fitness recovery: there are clear expression differences between wild-type and ancestral mutant worms, but expression patterns in evolved populations are overall much more similar to ancestral mutant worms than to wild-type worms (Chandler *et al.* 2012). This finding suggests that other changes underlie these apparently compensatory adaptations. Genetic variants segregating among wild isolates are known to modulate the phenotypic effects of these mutations (Chandler 2010), and some of these modifier alleles may have become fixed; new mutations in these populations might contribute as well.

Here, I use population-level, whole-genome resequencing in these populations to address the following questions: (i) to what degree have ancestral alleles become fixed, and how much recombination has occurred among these ancestral haplotypes? (ii) How similar are genomic patterns across experimental replicates? (iii) Is there evidence of any novel mutations? (iv) What selective scenarios best explain these outcomes? The results suggest that selection played an important role in generating the observed genomic outcomes, which here highly convergent across replicates and show similarities to evolve-and-resequence studies of isogenic microbial populations as well as outcrossing, genetically variable animal model systems. Moreover, the results also show clear parallels to patterns of genetic variation in natural populations of *C. elegans*—namely, strong selective sweeps with limited recombination among ancestral haplotypes.

## Materials and Methods

### Genome resequencing and analysis

A detailed description of the experimental evolution populations has been previously published (Chandler *et al.* 2012). In summary, the *C. elegans* strain CB5362 *tra-2(ar221)*II; *xol-1(y9)*X carries two mutations (in an N2 genetic background) that together result in an entirely XX population. These worms are hermaphrodites at cooler temperatures (<13°) and males at warmer temperatures (>24°). At the intermediate temperatures used in this experiment (16° and 18°), ancestral populations are a mix of males and hermaphrodites but with a high frequency of intersex phenotypes and strongly reduced fitness (Chandler *et al.* 2009, 2012). These mutations were introgressed into four additional wild-type genetic backgrounds (CB4856, AB1, MY2, and JU258; Chandler 2010; Chandler *et al.* 2012). Populations were founded with 10 hermaphrodites and 12 males with each ancestral genetic background; 10 replicate populations were evolved at 16° and another 10 at 18°. At generation 50 (g50), evolved populations displayed substantial phenotypic recovery (Chandler *et al.* 2012). Three populations showing strong evolutionary responses (18EE1, 18EE2, and 16EE6) were chosen for sequencing. In addition, I sequenced an ancestor sample consisting of DNA from pooled g1 worms of all three replicates.

Frozen samples of each population were thawed and grown at a permissive temperature (13°) for three to four generations before DNA isolation. For each population, hundreds of worms were washed *en masse* from Petri dishes in sterile water. DNA was extracted from each sample using a QIAGEN DNeasy Blood & Tissue Kit (QIAGEN, Valencia, CA) following the manufacturer’s instructions. The pooled ancestor sample and three evolved samples were submitted to the SUNY University at Buffalo Next-Generation Sequencing and Expression Analysis Core for sequencing on an Illumina HiSequation 2000 with single-end, 51-bp reads (1×51). In addition, I downloaded sequence data for wild-type strains CB4856, AB1, MY2, and JU258 from the NCBI Sequence Read Archive (accession numbers SRX219150, SRX128707, SRX218993, SRX218998, and SRX218971); these were paired-end reads (2x77 and 2x101). I mapped sequence reads to the reference *C. elegans* genome (WormBase release WS235) using BWA v0.7.5a (Li and Durbin 2009), allowing up to two mismatches for the 1×51 datasets, and using the default parameters with the BWA-MEM mapping algorithm for the other datasets. I called single-nucleotide polymorphisms (SNPs) using SAMTools v0.1.18 (Li *et al.* 2009), excluding sites with coverage greater than 100x to avoid calling false SNPs in paralogous repeats and restricted further analyses to SNPs with a minimum quality score of 30.

First, I wished to assess, for each position in the genome, which (if any) of the ancestral genetic backgrounds had become fixed. To do so, I identified SNP alleles unique to each of the five founder genetic backgrounds at nucleotide sites with at least 10× coverage in every background by using a custom R script. I then divided the genome into nonoverlapping 200-kb windows and calculated the average frequency of the alleles specific to each ancestral genetic background in each window, in each population.

Next, I looked for evidence of fixed *de novo* mutations, by identifying nucleotide substitutions that were nearly fixed (>90% frequency) in at least one evolved line but absent from the pooled ancestor and all the founder wild-type genetic backgrounds (and supported by at least 10× coverage in all cases).

### Simulation analyses

I used simulations to assess whether the observed outcomes were consistent with various levels of selection, numbers of fitness quantitative trait loci (QTL), and outcrossing rates. The source code for the simulation is archived on Dryad (DOI: 10.5061/dryad.603d2).

Simulations were written to reflect the design of the evolution experiment. In each run of the simulation, 20 populations were evolved for 50 generations each. The three populations with the greatest average fitness were selected and used to compute two test statistics: (i) *s*, the proportion of genomic positions in which the same allele was “dominant” (had the highest frequency) in all three populations; and (ii) *f*, the average proportion of sites fixed (in which the dominant allele has a frequency of at least 0.9) in each population. The expected distribution and 95% confidence intervals for each of these two statistics were computed using 200 replicate simulations for each set of parameter values (number of QTL, outcrossing rate, etc.). The observed values of these statistics (generated from the sequence data) were then compared with the expected distributions to generate a p-value for each scenario. A small p-value (< 0.05) for a given scenario would indicate a poor fit for the observed data, whereas a larger p-value for both statistics would indicate that the parameter values are consistent with the experimental results.

Each simulated population was initialized with 10 hermaphrodite and 12 male worms of each founder genetic background (N2, CB4856, AB1, MY2, and JU258), and subsequently expanded to a population size of 1000, with nonoverlapping generations. Each worm in the simulation carried a diploid XX genome, composed of six chromosome pairs of 69−105 “windows” each (corresponding to the 200-kb windows used in the analysis of the actual evolved genomes). Alternative population sizes of 200, 500, and 2000 also were tested and gave similar results (Supporting Information, Table S2).

For each generation *n*, the relative fitness of each simulated worm in the population was first calculated based on its genotype. A value of 1.0 was assigned as the default fitness. This fitness value was multiplied by the fitness assigned to each of the QTL alleles carried by the worm (see below for details on modeling QTL). The worm’s phenotypic sex was then chosen randomly based on the user-specified outcrossing rate.

Next, offspring genotypes for generation *n*+1 were generated one-by-one from the worm genotypes in generation *n*. To generate each offspring genotype, the first parent was selected randomly, weighted by relative fitness. If that parent was a hermaphrodite, the offspring genotype was generated by self-fertilization; if it was a male, a hermaphrodite mother was selected randomly, again weighted by relative fitness. Gamete genotypes were then generated by simulating meiosis. Each tetrad in the simulation underwent a single crossover during meiosis, mirroring the single crossover per chromosome pair observed in *C. elegans* (Hillers and Villeneuve 2003). After simulating crossing over, full gamete genotypes were obtained by randomly selecting a single chromatid for each of the six chromosome pairs making up the parental genotype. The complete offspring genotype was finally constructed by uniting the two gamete genotypes generated from the parent worms. This process was repeated until enough offspring genotypes had been generated to populate generation *n*+1. Again, in each replicate run of the simulation, this entire cycle was repeated for 50 generations in each of 20 populations, and the test statistics were computed for each of 200 replicates.

I ran the simulation with various sets of parameter values to assess which could best explain the observed results. Although the simulation was implemented in C++ for speed, the number of variables precluded an exhaustive search of parameter space for an optimal model fit. Therefore, I searched for parameter values that could produce a reasonable fit as measured by the two test statistics, focusing on three key factors: outcrossing rate, maximum relative fitness, and number and distribution of fitness QTL. I tested five outcrossing rate scenarios: constant rates of 0.01, 0.05, and 0.10, and two variable rates (initially 0.05 or 0.10, dropping to 0.01 at generation 25).

QTL were modeled by first selecting a value for the maximum relative fitness; five possible values for maximum relative fitness were tested: 1.1, 1.5, 2, 4, and 10. Then, for each maximum relative fitness value, various scenarios for the number and distribution of QTL were tested, based on the observed genotypes (see File S1 for details on how models describing the locations of QTL were generated based on observed outcomes). Finally, fitness values for QTL alleles were assigned such that the optimal genotype (*i.e.*, a simulated worm homozygous for all high-fitness alleles) would have the maximum relative fitness assigned earlier. For example, if the maximum relative fitness was 2.0, with two diploid QTL with equal effects, then the fitness value for each QTL would be 2^1/4^ ≈ 1.189; the fitness of the optimal genotype (say, *AABB* if *A* and *B* represent the high-fitness alleles at each locus) would therefore be 1.189^4^ ≈ 2. I also tested a null model with no fitness QTL (*i.e.*, neutral evolution).

Finally, in addition to the test statistics described previously, the simulation provided genome-wide allelic distributions for simulated evolved populations, so that the simulated outcomes could be qualitatively compared to the observed outcomes.

## Results

### Genomic patterns in experimental populations

I obtained a total of ~140 million single-end, 51-bp reads across the ancestral and evolved samples, resulting in at least ~9x genome-wide coverage for each population (Table S1). By using the same analysis pipeline with the publicly available sequence data for strains CB4856, AB1, MY2, and JU258, I obtained ~30x coverage for each of the wild-type ancestral genetic backgrounds. Raw sequence files generated in this study are available from the NCBI Sequence Read Archive under accession numbers SRX658438, SRX658572, SRX658573, and SRX658574. Processed data and analysis scripts are archived in Dryad (DOI: 10.5061/dryad.603d2).

**Figure 1:**
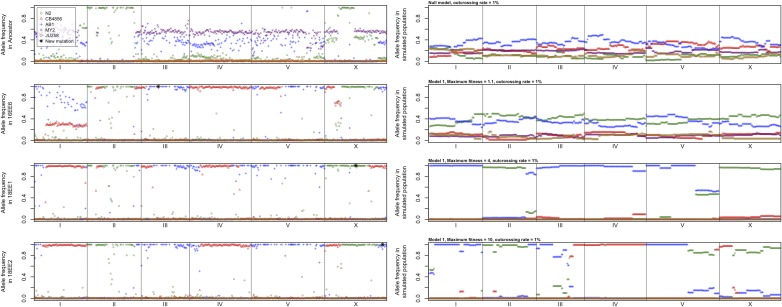
Frequencies of alleles specific to each founder genetic background in (left) a pooled ancestral sample and in the three sequenced evolved populations, and in (right) simulated populations for various parameter values. Each point represents a 200-kb genomic window. See text and Table 2 for details on simulation models.

Approximately 558,000 putative SNPs were detected, for an average density of one SNP every 180 bp. Of these, approximately 130,000 were specific to a single genetic background, for an average density of one diagnostic SNP per 750 bp, and 330 diagnostic SNPs per 200-kb window. Thus, power to detect heterozygosity within a given genomic window is fairly good because of the large number of diagnostic SNPs in each window, even though detecting heterozygosity at any individual SNP is low, given the modest sequencing depth. Indeed, substantial polymorphism was seen in the ancestor sample and on most of chromosome I in 16EE6. On the other hand, fixation or near-fixation of a single ancestral genetic background occurred throughout most of the genome in all the evolved lines; moreover, patterns of fixation were qualitatively similar in all three evolved lines (Figure 1). The large segments of contiguous ancestral backgrounds fixed on each chromosome in the evolved lines (most spanning several megabases) suggest that the genomic window size of 200 kb used here was small enough to capture the recombination events that have occurred in these populations (*i.e*., a single window is extremely unlikely to span more than one recombination breakpoint).

I found evidence for three fixed novel mutations in total: one coding mutation in line 16EE6 (a missense mutation in *nhl-2*), and one noncoding mutation each in lines 18EE1 and 18EE2 (Table 1). The use of Bowtie2 (Langmead and Salzberg 2012) as an alternative read aligner yielded nearly identical results for overall genomic composition and recovered the same three novel substitutions. Furthermore, all three mutations were confirmed using direct Sanger sequencing of PCR products (see File S2 and Table S3 for details). For the ancestral population, chromatograms only showed evidence of the reference allele, while only the mutant alleles were present in the respective evolved populations (Figure S2). These results suggest that the mutant alleles are indeed absent from the ancestral population and fixed in the evolved populations, or at least that the frequency of the minor allele is below the detection limit in each case.

**Table 1:** Candidate novel mutations in evolved lines

Chrom.	Pos.	Ref.	Alt.	Present in	Info.
III	4,895,774	A	C	16EE6	Leu→Arg at amino acid 862 in *nhl-2*
X	8,801,613	T	A	18EE1	Intergenic
X	16,501,330	C	T	18EE2	Intergenic

These candidate mutations were identified by searching for novel SNP alleles fixed in at least one evolved line (supported by at least 10× coverage) and absent from all ancestral sequences (again supported by at least 10× coverage). Chrom., chromosome; Pos., nucleotide position; Ref, reference allele; Alt., alternate allele; SNP, single-nucleotide polymorphism.

### Simulation results

Simulation analyses strongly rejected a null model of neutral evolution both quantitatively (*P* < 0.01; Table 2) and qualitatively (Figure 1). Models incorporating selection and low levels of outcrossing (1%), on the other hand, were sufficient to explain the shared patterns of fixation across the three replicate lines (Table 2), and gave genomic patterns qualitatively similar to those observed (Figure 1 and Figure S1). Results from simulations with alternative population sizes (200, 500, and 2000) were consistent overall (Table S2) in rejecting neutral evolution and supporting modest to large fitness gains for evolved worms, although greater outcrossing rates (5%) were also consistent with observed results in these cases.

**Table 2:** Results of simulations testing whether various selective scenarios are consistent with genomic outcomes observed in evolved lines

			1% Outcrossing		5% Outcrossing		10% Outcrossing		5% Outcrossing, Then 1% Outcrossing		10% Outcrossing, Then 1% Outcrossing	
Model	Max. Fitness	No. QTL	*p_s_* (95% CI)	*p_f_* (95% CI)	*p_s_* (95% CI)	*p_f_* (95% CI)	*p_s_* (95% CI)	*p_f_* (95% CI)	*p_s_* (95% CI)	*p_f_* (95% CI)	*p_s_* (95% CI)	*p_f_* (95% CI)
1	1.1	83	0.29 (0.00–0.83)	<0.01 (0.00–0.00)	0.10 (0.00–0.68)	<0.01 (0.00–0.00)	<0.01 (0.02–0.40)	<0.01 (0.00–0.00)	0.06 (0.00–0.58)	<0.01 (0.00–0.00)	<0.01 (0.02–0.40)	<0.01 (0.00–0.00)
1	1.5	83	0.28 (0.20–0.65)	<0.01 (0.00–0.13)	0.35 (0.21–0.64)	<0.01 (0.00–0.02)	0.35 (0.33–0.65)	<0.01 (0.00–0.00)	0.29 (0.25–0.65)	<0.01 (0.00–0.03)	0.36 (0.26–0.64)	<0.01 (0.00–0.01)
1	2	83	0.35 (0.20–0.73)	<0.01 (0.08–0.64)	0.48 (0.30–0.68)	<0.01 (0.01–0.21)	0.90 (0.38–0.76)	<0.01 (0.00–0.08)	0.31 (0.24–0.73)	<0.01 (0.02–0.35)	0.61 (0.29–0.68)	<0.01 (0.01–0.21)
1	4	83	**0.39 (0.25–0.65)**	**0.05 (0.31–0.85)**	0.99 (0.38–0.73)	<0.01 (0.22–0.65)	0.38 (0.44–0.79)	<0.01 (0.17–0.51)	0.91 (0.39–0.74)	<0.01 (0.26–0.74)	0.80 (0.41–0.75)	<0.01 (0.23–0.69)
1	10	83	**0.96 (0.40–0.71)**	**0.39 (0.53–0.96)**	0.38 (0.46–0.80)	0.04 (0.46–0.85)	0.12 (0.51–0.84)	0.01 (0.40–0.72)	**0.39 (0.47–0.79)**	**0.11 (0.54–0.88)**	**0.28 (0.51–0.79)**	**0.05 (0.50–0.84)**
2	1.1	22	0.96 (0.11–1.00)	<0.01 (0.00–0.00)	0.40 (0.14–0.78)	<0.01 (0.00–0.00)	0.13 (0.12–0.64)	<0.01 (0.00–0.00)	0.45 (0.11–0.79)	<0.01 (0.00–0.00)	0.14 (0.14–0.60)	<0.01 (0.00–0.00)
2	1.5	22	0.59 (0.45–0.77)	0.01 (0.21–0.78)	0.34 (0.47–0.80)	<0.01 (0.08–0.38)	0.20 (0.51–0.83)	<0.01 (0.01–0.15)	0.52 (0.43–0.80)	<0.01 (0.10–0.47)	0.53 (0.47–0.80)	<0.01 (0.04–0.27)
2	2	22	**0.32 (0.49–0.83)**	**0.43 (0.55–0.97)**	0.40 (0.46–0.81)	<0.01 (0.37–0.76)	0.28 (0.49–0.85)	<0.01 (0.23–0.51)	0.49 (0.46–0.80)	<0.01 (0.40–0.82)	0.41 (0.49–0.82)	<0.01 (0.29–0.66)
2	4	22	**0.46 (0.47–0.86)**	**0.82 (0.66–0.97)**	0.51 (0.48–0.79)	0.01 (0.46–0.80)	0.17 (0.52–0.82)	<0.01 (0.42–0.67)	0.66 (0.46–0.76)	<0.01 (0.47–0.81)	0.26 (0.51–0.79)	<0.01 (0.45–0.72)
2	10	22	**0.30 (0.47–0.86)**	**0.93 (0.66–0.98)**	0.28 (0.50–0.74)	0.01 (0.54–0.84)	0.13 (0.53–0.76)	<0.01 (0.52–0.75)	**0.45 (0.48–0.76)**	**0.12 (0.55–0.90)**	0.20 (0.52–0.76)	0.02 (0.54–0.83)
3	1.1	16	0.03 (0.60–1.00)	<0.01 (0.00–0.00)	0.36 (0.35–1.00)	<0.01 (0.00–0.00)	0.93 (0.27–0.84)	<0.01 (0.00–0.00)	0.15 (0.42–1.00)	<0.01 (0.00–0.00)	0.90 (0.30–0.93)	<0.01 (0.00–0.00)
3	1.5	16	0.67 (0.37–0.90)	<0.01 (0.26–0.57)	0.16 (0.49–0.92)	<0.01 (0.16–0.41)	0.02 (0.59–0.91)	<0.01 (0.07–0.20)	0.29 (0.44–0.91)	<0.01 (0.18–0.41)	0.21 (0.50–0.88)	<0.01 (0.10–0.28)
3	2	16	**0.75 (0.40–0.77)**	**0.07 (0.35–0.87)**	0.29 (0.48–0.84)	<0.01 (0.27–0.61)	0.13 (0.51–0.90)	<0.01 (0.23–0.47)	0.71 (0.38–0.80)	<0.01 (0.31–0.68)	0.45 (0.47–0.86)	<0.01 (0.27–0.55)
3	4	16	**0.84 (0.44–0.81)**	**0.42 (0.53–0.94)**	0.81 (0.46–0.77)	0.01 (0.45–0.78)	0.26 (0.49–0.80)	<0.01 (0.41–0.65)	1.00 (0.43–0.75)	0.03 (0.49–0.86)	0.71 (0.43–0.75)	0.01 (0.46–0.76)
3	10	16	**0.95 (0.42–0.77)**	**0.77 (0.62–0.98)**	0.51 (0.47–0.74)	0.01 (0.53–0.84)	0.18 (0.51–0.77)	<0.01 (0.50–0.79)	**0.72 (0.46–0.72)**	**0.20 (0.56–0.92)**	**0.50 (0.49–0.75)**	**0.07 (0.59–0.87)**
4	1.1	11	0.04 (0.63–1.00)	<0.01 (0.00–0.00)	0.19 (0.38–1.00)	<0.01 (0.00–0.00)	0.98 (0.24–0.91)	<0.01 (0.00–0.00)	0.19 (0.44–1.00)	<0.01 (0.00–0.00)	0.79 (0.21–0.94)	<0.01 (0.00–0.00)
4	1.5	11	0.63 (0.32–1.00)	<0.01 (0.22–0.62)	0.27 (0.40–0.96)	<0.01 (0.10–0.36)	0.09 (0.53–0.92)	<0.01 (0.01–0.13)	0.49 (0.40–0.97)	<0.01 (0.12–0.45)	0.32 (0.44–0.86)	<0.01 (0.04–0.24)
4	2	11	0.58 (0.29–0.70)	0.02 (0.34–0.82)	0.96 (0.34–0.76)	<0.01 (0.24–0.61)	0.55 (0.38–0.81)	<0.01 (0.17–0.40)	0.70 (0.31–0.74)	<0.01 (0.24–0.67)	0.75 (0.36–0.71)	<0.01 (0.19–0.51)
4	4	11	**0.49 (0.30–0.68)**	**0.40 (0.50–0.99)**	0.49 (0.33–0.66)	0.01 (0.39–0.76)	0.89 (0.41–0.72)	<0.01 (0.34–0.69)	0.28 (0.34–0.64)	0.04 (0.44–0.85)	0.65 (0.37–0.69)	0.01 (0.42–0.82)
4	10	11	**0.28 (0.31–0.63)**	**0.48 (0.55–0.97)**	**0.83 (0.37–0.69)**	**0.20 (0.54–0.90)**	0.86 (0.42–0.72)	0.03 (0.51–0.84)	**0.69 (0.40–0.67)**	**0.36 (0.56–0.95)**	**0.93 (0.38–0.70)**	**0.26 (0.60–0.94)**
Null	1	NA	0.01 (0.00–0.32)	0.01 (0.00–0.00)	<0.01 (0.00–0.23)	<0.01 (0.00–0.00)	<0.01 (0.00–0.16)	<0.01 (0.00–0.00)	<0.01 (0.00–0.20)	<0.01 (0.00–0.00)	<0.01 (0.00–0.10)	<0.01 (0.00–0.00)

In each model, the locations of fitness QTL were estimated from the observed genomic results. Each model was tested with various possible values for the maximum relative fitness and outcrossing rate. *p_s_* is the probability of the model resulting in a value of *s* (proportion of sites in which the same allele is dominant in all three evolved lines) at least as extreme as the observed value in 200 simulation replicates. *p_f_* is the probability of obtaining a value of *f* (average proportion of fixed sites across all three evolved lines) as extreme as the observed value. 95% confidence intervals for *s* and *f* are also given. The estimated observed values of *s* and *f* were 0.56 and 0.87, respectively. Models consistent with the observed data for both test statistics are in bold. CI, confidence interval; NA, not available; QTL, quantitative trait loci.

## Discussion

### Patterns of fixation

Earlier work has shown strong phenotypic changes in these *C. elegans* populations over the course of 50 generations of experimental evolution. In particular, although the *tra-2(ar221)*; *xol-1(y9)* genotype these populations were founded with results in high rates of infertility and intersexuality at the rearing temperatures used in this experiment, both of these mutant phenotypes are largely suppressed in the evolved lines (Chandler *et al.* 2012). The whole-genome resequencing data generated on this study shed light on the genomic changes that have accompanied this evolutionary response.

The high degree of fixation and similar allelic compositions of these evolved lines supports an important role for selection in generating the observed genomic outcomes. Indeed, simulations strongly reject neutral explanations for the observed results, instead supporting a role for selection (Figure 1 and Table 2). These simulations also suggest that the outcrossing rate in these populations was probably low (1–5%) and that the overall relative fitness has at least doubled in these populations, consistent with the strongly deleterious effects of the *tra-2(ar221)*; *xol-1(y9)* genotype and the large fitness recovery observed in the actual populations after 50 generations (Chandler *et al.* 2012). However, the simulations are not particularly informative with respect to the number of QTL; all four sets of QTL models produced similar outcomes for a given outcrossing rate and maximum fitness. Nevertheless, multiple loci are probably involved, as different genetic backgrounds became fixed in different parts of the genome in a repeatable manner (Figure 1).

In some ways, these results are similar to evolve-and-resequence studies in other animal models (Burke *et al.* 2010; Turner *et al.* 2011; Zhou *et al.* 2011; Remolina *et al.* 2012; Turner and Miller 2012; Tobler *et al.* 2014). For example, ancestral alleles, rather than new mutations, seem to have played an important role in the adaptive responses seen in these populations, based on the similarity of patterns of fixation across replicate populations (Figure 1). Indeed, evidence of just a single novel substitution was found in each population (Table 1), fewer than most studies involving microbes and one other study in *C. elegans* (Denver *et al.* 2010), although the sequencing approach used here could have missed some, such as substitutions in low-coverage regions or structural mutations. This paucity of new mutations may be explained by the presence of standing variation in the ancestral populations, which likely provided a rich pool of alleles for selection to act upon immediately. Indeed, a QTL mapping study showed that these genetic backgrounds harbor multiple polymorphisms modulating the effects of the *tra-2(ar221)*; *xol-1(y9)* genotype used in this experiment (Chandler 2010). However, these populations also evolved for fewer generations than most microbial experimental evolution studies (*e.g.*, Araya *et al.* 2010; Charusanti *et al.* 2010), which may have limited the opportunity for new mutations to accumulate.

In other aspects, on the other hand, these results resemble those of microbial studies. For instance, near-complete fixation, rather than moderate changes in allele frequencies, occurred throughout almost the entire genome in these populations (Figure 1). In addition, although some recombination among ancestral haplotypes did occur, entire chromosomal segments became fixed, suggesting that outcrossing rates in these populations were low, which was supported by simulations (Table 2), although segregating inversions could potentially explain this result too (Tobler *et al.* 2014).

These populations also display interesting differences from other studies looking at genetic diversity in experimentally evolved *C. elegans* populations. For instance, Chelo and Teotonio (2013) found much greater levels of diversity after 100 generations of experimental evolution in a novel environment. These differences can likely be explained by several factors. First, outcrossing rates in those populations were much higher (~20%; Teotonio *et al.* 2012). The lower outcrossing rates in my populations were likely caused by the mutant *tra-2(ar221)*; *xol-1(y9)* genotype, resulting in low-fitness males at these temperatures, and evolved populations are almost exclusively hermaphrodites (Chandler *et al.* 2012). In addition, the populations used by Chelo and Teotonio (2013) were initialized with variation from 16 wild genetic isolates using a more involved funnel cross, whereas my populations were established simply by placing several worms of each of five wild genetic backgrounds together in a single cross. The more controlled crossing scheme in their study therefore probably generated a much larger number of recombinant haplotypes at the very start of the experiment. Finally, the populations of Chelo and Teotonio (2013) also probably experienced weaker selection (selection coefficients around 2%, whereas fitness has probably at least doubled in this study). The stronger selection in this study is probably attributable to the deleterious effects of the mutant genotype fixed in these populations.

On the other hand, there are intriguing similarities to patterns of diversity seen in natural populations of *C. elegans*. Recent studies have found small numbers of haplotypes and strong linkage disequilibrium in wild isolates of *C. elegans* (Rockman and Kruglyak 2009; Andersen *et al.* 2012). These observations provide evidence for recent global selective sweeps, that outcrossing rates are low (Barrière and Félix 2005; 2007), and that selection against recombinant haplotypes drives effective population outcrossing rates even lower (Barrière and Félix 2007; Rockman and Kruglyak 2009). Similarly, the populations in this study showed evidence of low recombination and a selective sweep leading to the nearly complete fixation of a single haplotype in each population within 50 generations. Although the loss of diversity in these experimental populations is even more extreme than that seen in nature, the difference may be partially explained by the lack of metapopulation dynamics thought to characterize wild *C. elegans* populations (Barrière and Félix 2007); migration could reintroduce diversity into local subpopulations. Finally, the evolution of highly similar recombinant haplotypes in independent and completely isolated populations in this study suggests that shared haplotypes observed in distant localities in nature may not necessarily have arisen through migration, but perhaps instead through convergence.

In addition to the widespread fixation of ancestral alleles, one fixed novel mutation was detected in each population. The functional significance of these mutations is unclear. One is a missense mutation in *nhl-2*, which is involved in miRNA regulation (Hammell *et al.* 2009). A single intergenic substitution occurred in each of the other two lines, but these are not particularly close to any genes with obvious functions in sex determination or fertility, so whether they may influence fitness through effects on gene regulation remains to be tested. Alternatively, these mutations may be neutral, having simply hitchhiked on the recombinant chromosome that became fixed in each population.

### Selective pressures

Simulations strongly suggest that selection led to the observed genomic patterns, but they do not reveal the precise nature of those selective pressures. One possibility is that the multilocus genotypes observed in the evolved lines are the result of selection against deleterious combinations of epistatically interacting alleles segregating among the founder genetic backgrounds. Indeed, such incompatibilities are probably common (Corbett-Detig *et al.* 2014), and at least one good example is known in *C. elegans* (Seidel *et al.* 2008). Moreover, there is some evidence for selection against outcrossing in *C. elegans* in nature (Barrière and Félix 2007; Rockman and Kruglyak 2009). Although this scenario may explain some of the loci that became fixed in the evolved lines, I suspect that at least some of the fixed alleles in these populations were selected to compensate for the *tra-2(ar221)*; *xol-1(y9)* mutant genotype fixed in these populations. First, these mutations are highly deleterious and were fixed, so I expect selection to suppress their effects to be quite strong. Indeed, the nature of the phenotypic recovery seen in these lines—*e.g.*, the suppression of intersexed phenotypes—suggests that some of the increase in fitness in these populations is attributable to compensatory adaptation. Such compensatory evolution is probably common, as evidenced by a number of studies of both laboratory and natural systems (*e.g.*, (Moore *et al.* 2000; Estes and Lynch 2003; Kulathinal *et al.* 2004; Pischedda and Chippindale 2005; Harcombe *et al.* 2009; Stoebel *et al.* 2009).

In addition, the “ancestor” sequence data provide some suggestive clues. This pooled “ancestor” consisted of g1 worms that had undergone one generation of outcrossing before being frozen, thawed, and subsequently recovering for three to four generations. Thus, there was some, albeit limited, opportunity for selection to act before DNA isolation. During this process, worms were cultured at a permissive temperature (13°), at which the deleterious effects of these mutations are largely absent, so selection to compensate for their effects should have been weak, providing something of a negative control. Consistent with the idea that selection for compensatory adaptation explains allele frequencies in the evolved lines, genomic patterns in the evolved lines are distinct from those of the “ancestral” sample. These differences should be interpreted cautiously because of the small number of generations “ancestral” worms were reared at the permissive temperature and because of the potential for genetic drift in those populations after thawing. Nevertheless, they provide a possible hint that selection acts differently when the *tra-2(ar221)*; *xol-1(y9)* genotype’s deleterious effects are expressed, suggesting compensatory adaptation.

Unfortunately, generating a true negative control for this experiment would be difficult. For instance, populations seeded with the same wild genetic backgrounds but lacking the mutations would exhibit very different outcrossing rates, as males are typically maintained at relatively high frequencies in genetically mixed populations of *C. elegans* (Anderson *et al.* 2010; Teotonio *et al.* 2012). Thus, comparisons of genomic patterns between such wild-type “controls” and these experimental populations would be difficult to interpret.

### Fitness recovery in remaining populations and conclusions

The three evolved lines investigated here were chosen from a larger set of 20 because of their especially strong phenotypic recovery. But if the fitness increase in these lines was driven largely by pre-existing genetic variants, why was the evolutionary response in the other seventeen lines noticeably weaker? First, it is possible that the few detected novel substitutions did actually contribute to the strong response seen in these three lines, or that this strong recovery is caused by additional mutations that were undetected due to low sequencing coverage. An alternative explanation is that the stochastic loss of beneficial ancestral alleles early on in the experiment may have prevented most of the evolved lines from attaining such a robust recovery. Deeper sequencing of all the evolved lines, especially using paired-end sequence reads to help identify structural mutations, would help resolve this issue.

Most other evolve-and-resequence studies have focused on populations sitting at two ends of a continuum: either asexual, initially isogenic populations, or genetically variable, obligately outcrossing systems. In contrast, the populations in this study were seeded with a modest amount of genetic variation and exhibited a mixed mating system, similar to wild populations of *C. elegans*. As one might expect, genomic outcomes also were intermediate: although there was some recombination, ancestral variants became fixed in large genomic blocks, and there was evidence for fewer novel substitutions than in most microbial studies. This result is consistent with the low outcrossing rates and strong selective sweeps seen in natural populations of *C. elegans*. These results thus demonstrate the utility of the evolve-and-resequence approach in exploring how patterns of genomic diversity are shaped by factors such as selection, drift, mutation, and mating system.

## Supplementary Material

Supporting Information
