# Supplementary material for: Parallel Genome-Wide Fixation of Ancestral Alleles in Partially Outcrossing Experimental Populations of Caenorhabditis elegans
Source: G3 (Bethesda). 2014 Jul 1;4(9):1657–65. doi: 10.1534/g3.114.012914 (PMC4169157; doi:10.1534/g3.114.012914)
Supplement: Supporting Information [file supp_4_9_1657__index.html]

Parallel Genome-Wide Fixation of Ancestral Alleles in Partially Outcrossing Experimental Populations of Caenorhabditis elegans — Supporting Information 

# Parallel Genome-Wide Fixation of Ancestral Alleles in Partially Outcrossing Experimental Populations of *Caenorhabditis elegans*

## Supporting Information for Chandler, 2014

**Files in this Data Supplement:**

- Supporting Information - Files S1-S2, Tables S1-S3, and Figures S1-S2 (PDF, 472 KB)
- File S1 - Supplementary Methods (PDF, 134 KB)
- File S2 - Supplementary Results (PDF, 134 KB)
- Table S1 - Whole-genome re-sequencing datasets used in this study. (PDF, 103 KB)
- Table S2 - S2 Simulation results with alternative population sizes yield similar results, consistent with low outcrossing rates (1-5%), and moderate to large fitness differences between ancestral and evolved worms. (PDF, 127 KB)
- Table S3 - PCR and sequencing Primers used to confirm de novo mutations using Sanger sequencing. (PDF, 102 KB)
- Figure S1 - Frequencies of founding genetic background alleles in evolved lines and multiple simulated populations. (PDF, 653 KB)
- Figure S2 - Chromatograms from Sanger sequencing of PCR products confirming de novo mutations in evolved populations. (PDF, 216 KB)
