# Supplementary material for: Parallel Genome-Wide Fixation of Ancestral Alleles in Partially Outcrossing Experimental Populations of Caenorhabditis elegans
Source: G3 (Bethesda). 2014 Jul 1;4(9):1657–65. doi: 10.1534/g3.114.012914 (PMC4169157; doi:10.1534/g3.114.012914)
Supplement: Supporting Information [file supp_g3.114.012914_012914SI.pdf]

G3·Genes | Genomes | Genetics

Parallel genome-wide fixation of ancestral alleles in partially outcrossing experimental populations of *Caenorhabditis elegans*

Corresponding Author:
Christopher H. Chandler
Department of Biological Sciences
392 Shineman Center
SUNY Oswego
30 Centennial Drive
Oswego, NY 13126
Phone: 315-312-2774
E-mail: christopher.chandler@oswego.edu

DOI: 10.1534/g3.114.012914

File S1

Supplementary Methods

Generating models for quantitative trait loci affecting fitness—For each QTL model, QTL positions were assigned using the observed genomic outcomes using a custom R script. In the most complex model (model 1), a fitness QTL was placed at a given genomic window if a particular genetic background's alleles became fixed there in any of the evolved lines (with double or triple fitness benefits for alleles that became fixed in two or three evolved lines, respectively). However, to reduce the total number of QTLs, blocks of adjacent windows with the same observed outcome were combined into a single QTL. In model 2, only QTLs derived from blocks of at least 10 adjacent genomic windows were used. Model 3 was a reduced version of model 2, in which only QTL alleles that became fixed in at least two of the three evolved lines were kept. Model 4 was a further reduced version of model 3, in which equal fitness benefits were assigned to all QTL alleles (i.e., alleles that became fixed in all three evolved lines were assigned the same fitness values as alleles that became fixed in two of the three lines), and adjacent QTLs that became identical after simplifying fitness values were further collapsed. Again, the fitness effects of individual QTLs were assigned such that the optimal genotype would have the assigned maximum relative fitness (1.1, 1.5, 2, 4, or 10). Lastly, the null model contained no fitness QTLs, simulating neutral evolution. Files specifying the QTL locations and fitness values for each model are included with the simulation source code, along with an R script that generates these files from the observed outcomes.

File S2

Supplementary Results

Confirming fixed novel mutations with Sanger sequencing—I designed primers to amplify ~300-600 bp regions surrounding the three putative de novo mutations identified in the Illumina data (Supplementary Table 3). PCRs were run in 10 µL volumes containing 1x NH_4-based reaction buffer, 1.5 mM $MgCl_2$, 0.2 mM dNTPs, 0.5 µM primers, and 0.05 units Taq polymerase, with genomic DNA from ancestral and evolved populations as template. Thermal cycling consisted of an initial 3-minute denaturation at 95°C, followed by 35 cycles of 95°C denaturation for 1 minute, 50°C annealing for 1 minute, and extension at 72°C for 1 minute, plus a final extension step of 72°C for 5 minutes. Amplification of target regions was confirmed using agarose gel electrophoresis, and remaining PCR products were cleaned using ExoSAP-IT (Affymetrix) following the manufacturer's instructions. Sequencing mixtures consisting of 2.5 µL PCR product, 7.5 µL water, and 5 µL of 5 µM sequencing primer were prepared and submitted to GeneWiz (South Plainfield, NJ) for sequencing. For mutations 1 and 2, an internal sequencing primer was used; for mutation 3, the forward PCR primer was used for sequencing (Supplementary Table 3). Sanger sequencing confirmed the presence of each mutation in the evolved lines they were identified in with the Illumina data, as well as their absence in ancestral populations, at least at the detection threshold (Supplementary Figure 2).

Table S1 Whole-genome re-sequencing datasets used in this study. Numbers in "Source" column are NCBI SRA accession numbers.

Sample	Read size	Number of reads ($x10^6$)	Number of bases	Approximate coverage	Source
Ancestor	1x51 bp	54.3	2.8Gb	22x	This study
16EE6	1x51 bp	40.4	2.1Gb	14x	This study
18EE1	1x51 bp	22.9	1.2Gb	9x	This study
18EE2	1x51 bp	23.1	1.2Gb	9x	This study
CB4856	2x77, 2x101 bp	21.2, 83.0	3.2Gb, 16.6Gb	94x	SRX219150, SRX128707
AB1	2x101 bp	15.1	3.1Gb	25x	SRX218993
MY2	2x101 bp	15.3	3.1Gb	24x	SRX218998
JU258	2x101 bp	15.0	3.0Gb	23x	SRX218971

Table S2 Simulation results with alternative population sizes yield similar results, consistent with low outcrossing rates (1-5%), and moderate to large fitness differences between ancestral and evolved worms. As in Table 1, p_s is the probability of the model resulting in a value of *s* at least as extreme as the observed value in 200 simulation replicates, and p_f is the probability of obtaining a value of *f* as extreme as the observed value. 95% confidence intervals for *s* and *f* are also given. Models consistent with the observed data for both test statistics are in bold.

Model	Max. fitness	No. of QTLs	Population size = 200				Population size = 500				Population size = 2000			
			1% outcrossing		5% outcrossing		1% outcrossing		5% outcrossing		1% outcrossing		5% outcrossing	
			p_s (95% CI)	p_f (95% CI)	p_s (95% CI)	p_f (95% CI)	p_s (95% CI)	p_f (95% CI)	p_s (95% CI)	p_f (95% CI)	p_s (95% CI)	p_f (95% CI)	p_s (95% CI)	p_f (95% CI)
1	1.1	83	0.21 (0 - 0.94)	0.01 (0 - 0.3)	0.03 (0 - 0.5)	< 0.01 (0 - 0.12)	0.26 (0 - 0.81)	< 0.01 (0 - 0)	0.03 (0 - 0.52)	< 0.01 (0 - 0)	0.44 (0 - 0.88)	< 0.01 (0 - 0)	0.11 (0.02 - 0.61)	< 0.01 (0 - 0)
1	2	83	**0.11 (0.06 - 0.63)**	**0.95 (0.24 - 0.87)**	0.07 (0.12 - 0.58)	0.04 (0.11 - 0.53)	**0.2 (0.15 - 0.64)**	**0.38 (0.13 - 0.75)**	0.21 (0.23 - 0.62)	< 0.01 (0.03 - 0.38)	0.49 (0.22 - 0.73)	< 0.01 (0.04 - 0.41)	0.93 (0.32 - 0.75)	< 0.01 (0 - 0.14)
1	4	83	**0.16 (0.17 - 0.65)**	**0.19 (0.44 - 0.98)**	**0.17 (0.22 - 0.61)**	**0.99 (0.33 - 0.82)**	**0.26 (0.22 - 0.68)**	**0.71 (0.33 - 0.94)**	**0.69 (0.32 - 0.7)**	**0.39 (0.25 - 0.7)**	**0.44 (0.27 - 0.66)**	**0.63 (0.23 - 0.77)**	**0.62 (0.4 - 0.79)**	**0.17 (0.2 - 0.67)**
1	10	83	**0.17 (0.21 - 0.6)**	**0.06 (0.53 - 0.97)**	**0.65 (0.37 - 0.68)**	**0.11 (0.54 - 0.91)**	**0.66 (0.34 - 0.68)**	**0.11 (0.49 - 0.98)**	**0.49 (0.46 - 0.76)**	**0.34 (0.47 - 0.87)**	**0.56 (0.44 - 0.74)**	**0.12 (0.53 - 0.95)**	**0.14 (0.52 - 0.82)**	**0.33 (0.49 - 0.84)**
2	1.1	22	0.5 (0 - 1)	< 0.01 (0 - 0.33)	0.11 (0 - 0.67)	< 0.01 (0 - 0.19)	0.63 (0.02 - 1)	< 0.01 (0 - 0.01)	0.2 (0.03 - 0.71)	< 0.01 (0 - 0)	0.67 (0.25 - 1)	< 0.01 (0 - 0)	0.93 (0.25 - 0.9)	< 0.01 (0 - 0)
2	2	22	0.97 (0.36 - 0.74)	0.02 (0.58 - 0.99)	**0.98 (0.4 - 0.76)**	**0.38 (0.45 - 0.87)**	**0.59 (0.44 - 0.79)**	**0.06 (0.55 - 0.97)**	**0.69 (0.43 - 0.8)**	**0.73 (0.42 - 0.81)**	**0.22 (0.5 - 0.83)**	**0.08 (0.56 - 0.93)**	**0.21 (0.49 - 0.85)**	**0.42 (0.35 - 0.67)**
2	4	22	0.67 (0.42 - 0.79)	< 0.01 (0.73 - 0.99)	**0.94 (0.43 - 0.78)**	**0.06 (0.55 - 0.9)**	0.37 (0.46 - 0.8)	< 0.01 (0.69 - 0.98)	**0.55 (0.46 - 0.78)**	**0.25 (0.48 - 0.87)**	0.28 (0.48 - 0.86)	< 0.01 (0.63 - 0.97)	**0.28 (0.48 - 0.8)**	**0.97 (0.44 - 0.72)**
2	10	22	0.36 (0.46 - 0.84)	< 0.01 (0.75 - 1)	0.93 (0.43 - 0.75)	< 0.01 (0.65 - 0.94)	0.39 (0.49 - 0.85)	< 0.01 (0.71 - 0.99)	0.52 (0.46 - 0.77)	0.03 (0.57 - 0.88)	0.34 (0.47 - 0.84)	0.03 (0.6 - 0.97)	**0.21 (0.51 - 0.78)**	**0.12 (0.52 - 0.82)**
3	1.1	16	**0.55 (0.27 - 1)**	**0.09 (0 - 0.6)**	0.79 (0.15 - 0.95)	< 0.01 (0 - 0.23)	0.23 (0.38 - 1)	< 0.01 (0 - 0.12)	0.71 (0.26 - 0.98)	< 0.01 (0 - 0.02)	< 0.01 (0.8 - 1)	< 0.01 (0 - 0)	0.05 (0.56 - 1)	< 0.01 (0 - 0)
3	2	16	**0.71 (0.32 - 0.69)**	**0.24 (0.43 - 0.97)**	**0.94 (0.36 - 0.78)**	**0.91 (0.41 - 0.81)**	**0.96 (0.38 - 0.77)**	**0.47 (0.45 - 0.87)**	**0.74 (0.4 - 0.8)**	**0.49 (0.33 - 0.71)**	**0.57 (0.45 - 0.79)**	**0.83 (0.37 - 0.88)**	0.22 (0.46 - 0.88)	0.02 (0.28 - 0.55)
3	4	16	0.86 (0.41 - 0.74)	< 0.01 (0.68 - 0.99)	**0.93 (0.37 - 0.75)**	**0.09 (0.54 - 0.92)**	0.75 (0.4 - 0.8)	< 0.01 (0.62 - 0.98)	**0.77 (0.44 - 0.77)**	**0.24 (0.5 - 0.85)**	**0.79 (0.4 - 0.82)**	**0.1 (0.53 - 0.97)**	**0.63 (0.45 - 0.78)**	**0.92 (0.43 - 0.74)**
3	10	16	0.75 (0.39 - 0.79)	< 0.01 (0.77 - 0.99)	0.85 (0.41 - 0.77)	< 0.01 (0.66 - 0.94)	0.78 (0.41 - 0.81)	< 0.01 (0.68 - 0.99)	**0.86 (0.43 - 0.72)**	**0.06 (0.56 - 0.85)**	**0.93 (0.41 - 0.72)**	**0.05 (0.56 - 0.94)**	**0.45 (0.48 - 0.73)**	**0.12 (0.53 - 0.9)**
4	1.1	11	0.52 (0.3 - 1)	0.03 (0 - 0.51)	**0.9 (0.11 - 0.94)**	**< 0.01 (0 - 0.23)**	0.22 (0.38 - 1)	< 0.01 (0 - 0.15)	0.77 (0.19 - 1)	< 0.01 (0 - 0.02)	< 0.01 (0.93 - 1)	< 0.01 (0 - 0)	0.01 (0.6 - 1)	< 0.01 (0 - 0)
4	2	11	**0.24 (0.19 - 0.67)**	**0.42 (0.39 - 0.92)**	**0.32 (0.27 - 0.7)**	**0.87 (0.34 - 0.77)**	**0.34 (0.25 - 0.65)**	**0.75 (0.35 - 0.9)**	**0.72 (0.27 - 0.72)**	**0.25 (0.27 - 0.65)**	**0.76 (0.33 - 0.72)**	**0.62 (0.3 - 0.79)**	0.77 (0.38 - 0.8)	0.01 (0.23 - 0.54)
4	4	11	0.36 (0.22 - 0.7)	0.01 (0.62 - 0.99)	**0.41 (0.3 - 0.65)**	**0.09 (0.54 - 0.91)**	**0.49 (0.26 - 0.68)**	**0.05 (0.55 - 0.98)**	**0.51 (0.33 - 0.66)**	**0.74 (0.41 - 0.79)**	**0.4 (0.29 - 0.66)**	**0.42 (0.4 - 0.94)**	**0.7 (0.37 - 0.69)**	**0.75 (0.36 - 0.73)**
4	10	11	0.46 (0.29 - 0.69)	< 0.01 (0.68 - 0.99)	0.49 (0.34 - 0.68)	< 0.01 (0.64 - 0.93)	0.27 (0.32 - 0.65)	< 0.01 (0.63 - 0.98)	**0.46 (0.38 - 0.66)**	**0.08 (0.55 - 0.9)**	0.47 (0.36 - 0.66)	0.03 (0.58 - 0.97)	**0.76 (0.4 - 0.68)**	**0.05 (0.55 - 0.9)**
Null	1	NA	0.01 (0 - 0.27)	< 0.01 (0 - 0.05)	< 0.01 (0 - 0.23)	< 0.01 (0 - 0.04)	0.01 (0 - 0.3)	< 0.01 (0 - 0)	< 0.01 (0 - 0.15)	< 0.01 (0 - 0)	< 0.01 (0 - 0.24)	< 0.01 (0 - 0)	< 0.01 (0 - 0.13)	< 0.01 (0 - 0)

Table S3 PCR and sequencing Primers used to confirm de novo mutations using Sanger sequencing.

Mutation Number	Mutation Position	PCR Product Size (bp)	Primers
1	III: 4,895,774	571	F: ATGTTGTCCTTCGGGTGAGA<br>R: GGACAATTGTGCTTCCCAAG<br>Seq: TTGGACACAATGATGCTTGAA
2	X: 8,801,613	564	F: AATCCATTCGCTGGAGTAGC<br>R: TGAATCAGTCCCGAAGAACC<br>Seq: ACCAGGAGCAAATTGGAAGA
3	X: 16,501,330	366	F: TCTTCCGGGTAGAGATAAACGA<br>R: CACTGTTGGGTGCTTTTCAA

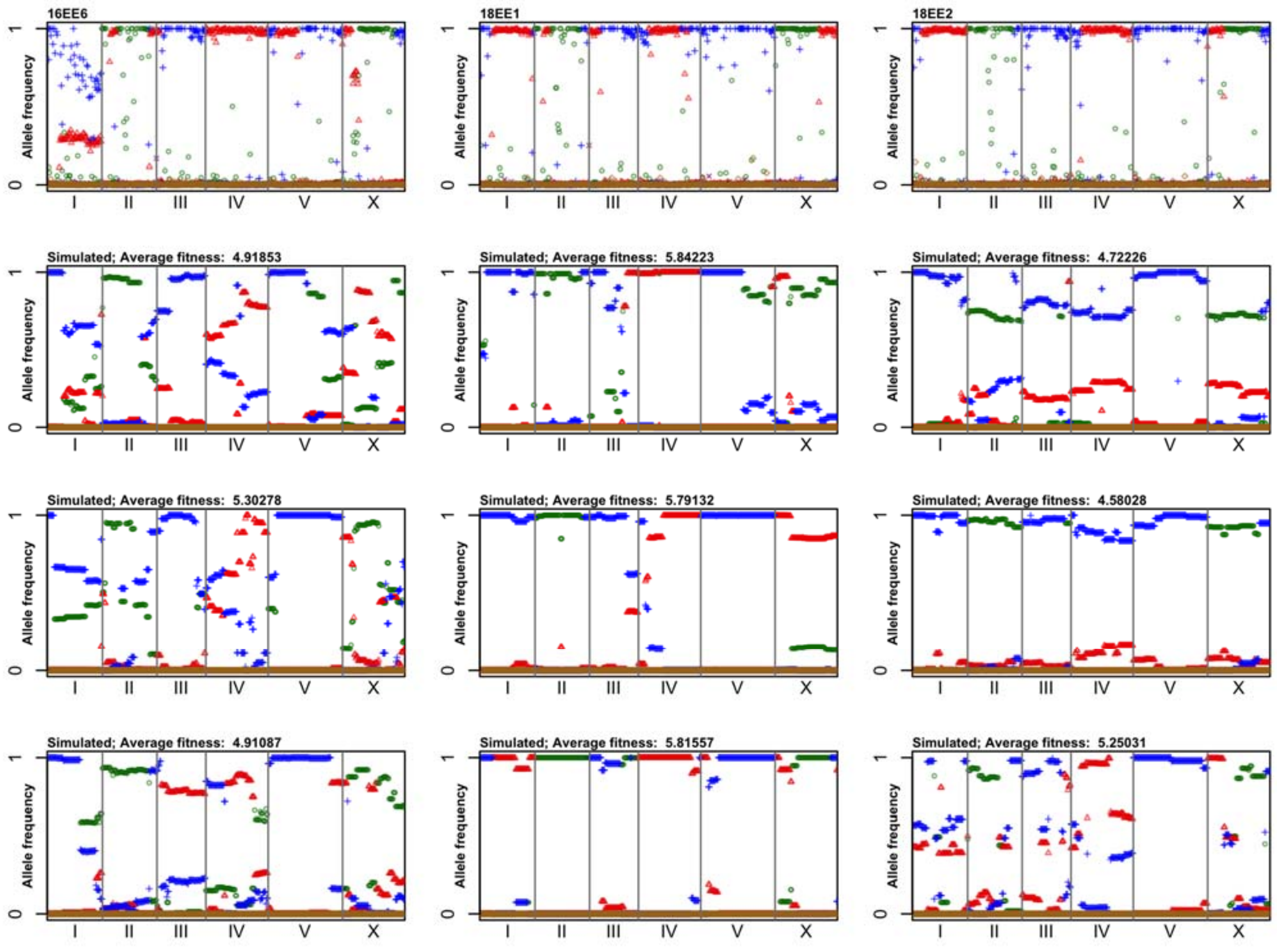

Figure S1 Frequencies of founding genetic background alleles in evolved lines and multiple simulated populations. Top three panels: actual experimental evolution lines. Remaining panels: simulated populations (model 1 with a maximum relative fitness of 10 and a 1% outcrossing rate). Each panel represents a single population. Point symbols/colors indicate the founder genetic backgrounds as in Figure 1.

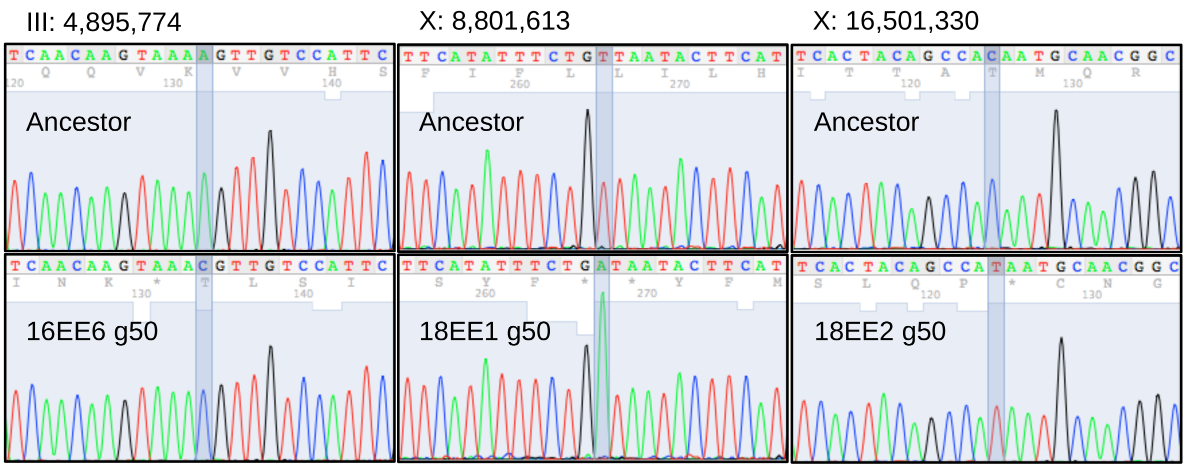

Figure S2 Chromatograms from Sanger sequencing of PCR products confirming de novo mutations in evolved populations. The mutated bases are highlighted.
