# Supplementary material for: Parallel Genome-Wide Fixation of Ancestral Alleles in Partially Outcrossing Experimental Populations of Caenorhabditis elegans
Source: G3 (Bethesda). 2014 Jul 1;4(9):1657–65. doi: 10.1534/g3.114.012914 (PMC4169157; doi:10.1534/g3.114.012914)
Supplement: Supporting Information [file supp_g3.114.012914_FileS1.pdf]

File S1

Supplementary Methods

Generating models for quantitative trait loci affecting fitness—For each QTL model, QTL positions were assigned using the observed genomic outcomes using a custom R script. In the most complex model (model 1), a fitness QTL was placed at a given genomic window if a particular genetic background's alleles became fixed there in any of the evolved lines (with double or triple fitness benefits for alleles that became fixed in two or three evolved lines, respectively). However, to reduce the total number of QTLs, blocks of adjacent windows with the same observed outcome were combined into a single QTL. In model 2, only QTLs derived from blocks of at least 10 adjacent genomic windows were used. Model 3 was a reduced version of model 2, in which only QTL alleles that became fixed in at least two of the three evolved lines were kept. Model 4 was a further reduced version of model 3, in which equal fitness benefits were assigned to all QTL alleles (i.e., alleles that became fixed in all three evolved lines were assigned the same fitness values as alleles that became fixed in two of the three lines), and adjacent QTLs that became identical after simplifying fitness values were further collapsed. Again, the fitness effects of individual QTLs were assigned such that the optimal genotype would have the assigned maximum relative fitness (1.1, 1.5, 2, 4, or 10). Lastly, the null model contained no fitness QTLs, simulating neutral evolution. Files specifying the QTL locations and fitness values for each model are included with the simulation source code, along with an R script that generates these files from the observed outcomes.
