# Supplementary material for: Parallel Genome-Wide Fixation of Ancestral Alleles in Partially Outcrossing Experimental Populations of Caenorhabditis elegans
Source: G3 (Bethesda). 2014 Jul 1;4(9):1657–65. doi: 10.1534/g3.114.012914 (PMC4169157; doi:10.1534/g3.114.012914)
Supplement: Supporting Information [file supp_g3.114.012914_FileS2.pdf]

## File S2

## Supplementary Results

*Confirming fixed novel mutations with Sanger sequencing*—I designed primers to amplify ~300-600 bp regions surrounding the three putative de novo mutations identified in the Illumina data (Supplementary Table 3). PCRs were run in 10 µL volumes containing 1x $NH_4$-based reaction buffer, 1.5 mM $MgCl_2$, 0.2 mM dNTPs, 0.5 µM primers, and 0.05 units Taq polymerase, with genomic DNA from ancestral and evolved populations as template. Thermal cycling consisted of an initial 3-minute denaturation at 95°C, followed by 35 cycles of 95°C denaturation for 1 minute, 50°C annealing for 1 minute, and extension at 72°C for 1 minute, plus a final extension step of 72°C for 5 minutes. Amplification of target regions was confirmed using agarose gel electrophoresis, and remaining PCR products were cleaned using ExoSAP-IT (Affymetrix) following the manufacturer's instructions. Sequencing mixtures consisting of 2.5 µL PCR product, 7.5 µL water, and 5 µL of 5 µM sequencing primer were prepared and submitted to GeneWiz (South Plainfield, NJ) for sequencing. For mutations 1 and 2, an internal sequencing primer was used; for mutation 3, the forward PCR primer was used for sequencing (Supplementary Table 3). Sanger sequencing confirmed the presence of each mutation in the evolved lines they were identified in with the Illumina data, as well as their absence in ancestral populations, at least at the detection threshold (Supplementary Figure 2).
