# Supplementary material for: Parallel Genome-Wide Fixation of Ancestral Alleles in Partially Outcrossing Experimental Populations of Caenorhabditis elegans
Source: G3 (Bethesda). 2014 Jul 1;4(9):1657–65. doi: 10.1534/g3.114.012914 (PMC4169157; doi:10.1534/g3.114.012914)
Supplement: Supporting Information [file supp_g3.114.012914_TableS1.pdf]

Table S1 Whole-genome re-sequencing datasets used in this study. Numbers in "Source" column are NCBI SRA accession numbers.

Sample	Read size	Number of reads ($x10^6$)	Number of bases	Approximate coverage	Source
Ancestor	1x51 bp	54.3	2.8Gb	22x	This study
16EE6	1x51 bp	40.4	2.1Gb	14x	This study
18EE1	1x51 bp	22.9	1.2Gb	9x	This study
18EE2	1x51 bp	23.1	1.2Gb	9x	This study
CB4856	2x77, 2x101 bp	21.2, 83.0	3.2Gb, 16.6Gb	94x	SRX219150, SRX128707
AB1	2x101 bp	15.1	3.1Gb	25x	SRX218993
MY2	2x101 bp	15.3	3.1Gb	24x	SRX218998
JU258	2x101 bp	15.0	3.0Gb	23x	SRX218971
