# Supplementary material for: Parallel Genome-Wide Fixation of Ancestral Alleles in Partially Outcrossing Experimental Populations of Caenorhabditis elegans
Source: G3 (Bethesda). 2014 Jul 1;4(9):1657–65. doi: 10.1534/g3.114.012914 (PMC4169157; doi:10.1534/g3.114.012914)
Supplement: Supporting Information [file supp_g3.114.012914_TableS2.pdf]

Table S2 Simulation results with alternative population sizes yield similar results, consistent with low outcrossing rates (1-5%), and moderate to large fitness differences between ancestral and evolved worms. As in Table 1, p_s is the probability of the model resulting in a value of *s* at least as extreme as the observed value in 200 simulation replicates, and p_f is the probability of obtaining a value of *f* as extreme as the observed value. 95% confidence intervals for *s* and *f* are also given. Models consistent with the observed data for both test statistics are in bold.

Model	Max. fitness	No. of QTLs	Population size = 200				Population size = 500				Population size = 2000			
			1% outcrossing		5% outcrossing		1% outcrossing		5% outcrossing		1% outcrossing		5% outcrossing	
			p_s (95% CI)	p_f (95% CI)	p_s (95% CI)	p_f (95% CI)	p_s (95% CI)	p_f (95% CI)	p_s (95% CI)	p_f (95% CI)	p_s (95% CI)	p_f (95% CI)	p_s (95% CI)	p_f (95% CI)
1	1.1	83	0.21 (0 - 0.94)	0.01 (0 - 0.3)	0.03 (0 - 0.5)	< 0.01 (0 - 0.12)	0.26 (0 - 0.81)	< 0.01 (0 - 0)	0.03 (0 - 0.52)	< 0.01 (0 - 0)	0.44 (0 - 0.88)	< 0.01 (0 - 0)	0.11 (0.02 - 0.61)	< 0.01 (0 - 0)
1	2	83	**0.11 (0.06 - 0.63)**	**0.95 (0.24 - 0.87)**	0.07 (0.12 - 0.58)	0.04 (0.11 - 0.53)	**0.2 (0.15 - 0.64)**	**0.38 (0.13 - 0.75)**	0.21 (0.23 - 0.62)	< 0.01 (0.03 - 0.38)	0.49 (0.22 - 0.73)	< 0.01 (0.04 - 0.41)	0.93 (0.32 - 0.75)	< 0.01 (0 - 0.14)
1	4	83	**0.16 (0.17 - 0.65)**	**0.19 (0.44 - 0.98)**	**0.17 (0.22 - 0.61)**	**0.99 (0.33 - 0.82)**	**0.26 (0.22 - 0.68)**	**0.71 (0.33 - 0.94)**	**0.69 (0.32 - 0.7)**	**0.39 (0.25 - 0.7)**	**0.44 (0.27 - 0.66)**	**0.63 (0.23 - 0.77)**	**0.62 (0.4 - 0.79)**	**0.17 (0.2 - 0.67)**
1	10	83	**0.17 (0.21 - 0.6)**	**0.06 (0.53 - 0.97)**	**0.65 (0.37 - 0.68)**	**0.11 (0.54 - 0.91)**	**0.66 (0.34 - 0.68)**	**0.11 (0.49 - 0.98)**	**0.49 (0.46 - 0.76)**	**0.34 (0.47 - 0.87)**	**0.56 (0.44 - 0.74)**	**0.12 (0.53 - 0.95)**	**0.14 (0.52 - 0.82)**	**0.33 (0.49 - 0.84)**
2	1.1	22	0.5 (0 - 1)	< 0.01 (0 - 0.33)	0.11 (0 - 0.67)	< 0.01 (0 - 0.19)	0.63 (0.02 - 1)	< 0.01 (0 - 0.01)	0.2 (0.03 - 0.71)	< 0.01 (0 - 0)	0.67 (0.25 - 1)	< 0.01 (0 - 0)	0.93 (0.25 - 0.9)	< 0.01 (0 - 0)
2	2	22	0.97 (0.36 - 0.74)	0.02 (0.58 - 0.99)	**0.98 (0.4 - 0.76)**	**0.38 (0.45 - 0.87)**	**0.59 (0.44 - 0.79)**	**0.06 (0.55 - 0.97)**	**0.69 (0.43 - 0.8)**	**0.73 (0.42 - 0.81)**	**0.22 (0.5 - 0.83)**	**0.08 (0.56 - 0.93)**	**0.21 (0.49 - 0.85)**	**0.42 (0.35 - 0.67)**
2	4	22	0.67 (0.42 - 0.79)	< 0.01 (0.73 - 0.99)	**0.94 (0.43 - 0.78)**	**0.06 (0.55 - 0.9)**	0.37 (0.46 - 0.8)	< 0.01 (0.69 - 0.98)	**0.55 (0.46 - 0.78)**	**0.25 (0.48 - 0.87)**	0.28 (0.48 - 0.86)	< 0.01 (0.63 - 0.97)	**0.28 (0.48 - 0.8)**	**0.97 (0.44 - 0.72)**
2	10	22	0.36 (0.46 - 0.84)	< 0.01 (0.75 - 1)	0.93 (0.43 - 0.75)	< 0.01 (0.65 - 0.94)	0.39 (0.49 - 0.85)	< 0.01 (0.71 - 0.99)	0.52 (0.46 - 0.77)	0.03 (0.57 - 0.88)	0.34 (0.47 - 0.84)	0.03 (0.6 - 0.97)	**0.21 (0.51 - 0.78)**	**0.12 (0.52 - 0.82)**
3	1.1	16	**0.55 (0.27 - 1)**	**0.09 (0 - 0.6)**	0.79 (0.15 - 0.95)	< 0.01 (0 - 0.23)	0.23 (0.38 - 1)	< 0.01 (0 - 0.12)	0.71 (0.26 - 0.98)	< 0.01 (0 - 0.02)	< 0.01 (0.8 - 1)	< 0.01 (0 - 0)	0.05 (0.56 - 1)	< 0.01 (0 - 0)
3	2	16	**0.71 (0.32 - 0.69)**	**0.24 (0.43 - 0.97)**	**0.94 (0.36 - 0.78)**	**0.91 (0.41 - 0.81)**	**0.96 (0.38 - 0.77)**	**0.47 (0.45 - 0.87)**	**0.74 (0.4 - 0.8)**	**0.49 (0.33 - 0.71)**	**0.57 (0.45 - 0.79)**	**0.83 (0.37 - 0.88)**	0.22 (0.46 - 0.88)	0.02 (0.28 - 0.55)
3	4	16	0.86 (0.41 - 0.74)	< 0.01 (0.68 - 0.99)	**0.93 (0.37 - 0.75)**	**0.09 (0.54 - 0.92)**	0.75 (0.4 - 0.8)	< 0.01 (0.62 - 0.98)	**0.77 (0.44 - 0.77)**	**0.24 (0.5 - 0.85)**	**0.79 (0.4 - 0.82)**	**0.1 (0.53 - 0.97)**	**0.63 (0.45 - 0.78)**	**0.92 (0.43 - 0.74)**
3	10	16	0.75 (0.39 - 0.79)	< 0.01 (0.77 - 0.99)	0.85 (0.41 - 0.77)	< 0.01 (0.66 - 0.94)	0.78 (0.41 - 0.81)	< 0.01 (0.68 - 0.99)	**0.86 (0.43 - 0.72)**	**0.06 (0.56 - 0.85)**	**0.93 (0.41 - 0.72)**	**0.05 (0.56 - 0.94)**	**0.45 (0.48 - 0.73)**	**0.12 (0.53 - 0.9)**
4	1.1	11	0.52 (0.3 - 1)	0.03 (0 - 0.51)	**0.9 (0.11 - 0.94)**	**< 0.01 (0 - 0.23)**	0.22 (0.38 - 1)	< 0.01 (0 - 0.15)	0.77 (0.19 - 1)	< 0.01 (0 - 0.02)	< 0.01 (0.93 - 1)	< 0.01 (0 - 0)	0.01 (0.6 - 1)	< 0.01 (0 - 0)
4	2	11	**0.24 (0.19 - 0.67)**	**0.42 (0.39 - 0.92)**	**0.32 (0.27 - 0.7)**	**0.87 (0.34 - 0.77)**	**0.34 (0.25 - 0.65)**	**0.75 (0.35 - 0.9)**	**0.72 (0.27 - 0.72)**	**0.25 (0.27 - 0.65)**	**0.76 (0.33 - 0.72)**	**0.62 (0.3 - 0.79)**	0.77 (0.38 - 0.8)	0.01 (0.23 - 0.54)
4	4	11	0.36 (0.22 - 0.7)	0.01 (0.62 - 0.99)	**0.41 (0.3 - 0.65)**	**0.09 (0.54 - 0.91)**	**0.49 (0.26 - 0.68)**	**0.05 (0.55 - 0.98)**	**0.51 (0.33 - 0.66)**	**0.74 (0.41 - 0.79)**	**0.4 (0.29 - 0.66)**	**0.42 (0.4 - 0.94)**	**0.7 (0.37 - 0.69)**	**0.75 (0.36 - 0.73)**
4	10	11	0.46 (0.29 - 0.69)	< 0.01 (0.68 - 0.99)	0.49 (0.34 - 0.68)	< 0.01 (0.64 - 0.93)	0.27 (0.32 - 0.65)	< 0.01 (0.63 - 0.98)	**0.46 (0.38 - 0.66)**	**0.08 (0.55 - 0.9)**	0.47 (0.36 - 0.66)	0.03 (0.58 - 0.97)	**0.76 (0.4 - 0.68)**	**0.05 (0.55 - 0.9)**
Null	1	NA	0.01 (0 - 0.27)	< 0.01 (0 - 0.05)	< 0.01 (0 - 0.23)	< 0.01 (0 - 0.04)	0.01 (0 - 0.3)	< 0.01 (0 - 0)	< 0.01 (0 - 0.15)	< 0.01 (0 - 0)	< 0.01 (0 - 0.24)	< 0.01 (0 - 0)	< 0.01 (0 - 0.13)	< 0.01 (0 - 0)
