# Supplementary material for: Parallel Genome-Wide Fixation of Ancestral Alleles in Partially Outcrossing Experimental Populations of Caenorhabditis elegans
Source: G3 (Bethesda). 2014 Jul 1;4(9):1657–65. doi: 10.1534/g3.114.012914 (PMC4169157; doi:10.1534/g3.114.012914)
Supplement: Supporting Information [file supp_g3.114.012914_TableS3.pdf]

Table S3 PCR and sequencing Primers used to confirm de novo mutations using Sanger sequencing.

Mutation Number	Mutation Position	PCR Product Size (bp)	Primers
1	III: 4,895,774	571	F: ATGTTGTCCTTCGGGTGAGA<br>R: GGACAATTGTGCTTCCCAAG<br>Seq: TTGGACACAATGATGCTTGAA
2	X: 8,801,613	564	F: AATCCATTCGCTGGAGTAGC<br>R: TGAATCAGTCCCGAAGAACC<br>Seq: ACCAGGAGCAAATTGGAAGA
3	X: 16,501,330	366	F: TCTTCCGGGTAGAGATAAACGA<br>R: CACTGTTGGGTGCTTTTCAA
