# Supplementary material for: Parallel Genome-Wide Fixation of Ancestral Alleles in Partially Outcrossing Experimental Populations of Caenorhabditis elegans
Source: G3 (Bethesda). 2014 Jul 1;4(9):1657–65. doi: 10.1534/g3.114.012914 (PMC4169157; doi:10.1534/g3.114.012914)
Supplement: Supporting Information [file supp_g3.114.012914_FigureS1.pdf]

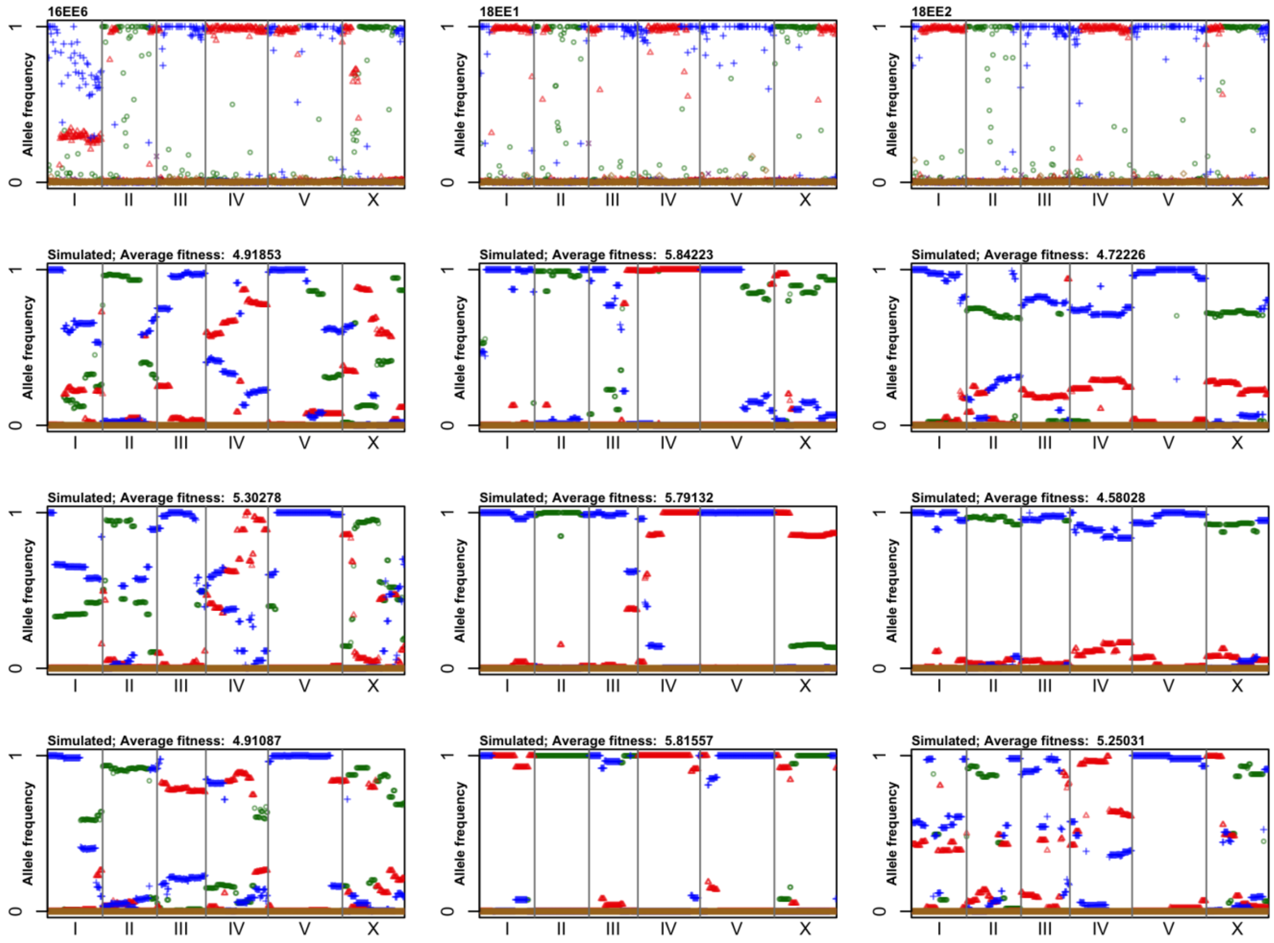

Figure S1 Frequencies of founding genetic background alleles in evolved lines and multiple simulated populations. Top three panels: actual experimental evolution lines. Remaining panels: simulated populations (model 1 with a maximum relative fitness of 10 and a 1% outcrossing rate). Each panel represents a single population. Point symbols/colors indicate the founder genetic backgrounds as in Figure 1.
